# Abdominal normothermic regional perfusion is associated with improved graft survival in liver transplantation from circulatory death donors

**DOI:** 10.3389/ti.2026.16004

**Published:** 2026-08-26

**Authors:** Giulia Magini, Hannah Wozniak, Antonia Schafer, Beat Moeckli, Vanessa Banz, Julien Vionnet, Ansgar Deibel, Jose Oberholzer, Pascale Tinguely, Nicolas Goossens, Andrea Peloso, Mirjam Korner, Franz Immer, Riccardo De Carlis, Christian Toso, Benjamin Assouline, Karim Bendjelid, Philippe Compagnon, Raphael Giraud

**Affiliations:** 1 Division of Transplantation, Department of Surgery, Geneva University Hospitals, Geneva, Switzerland; 2 Intensive Care Division, Geneva University Hospitals, Geneva, Switzerland; 3 Transplantation Immunology Unit and National Reference Laboratory for Histocompatibility, Department of Diagnostic, Geneva University Hospitals, Geneva, Switzerland; 4 Division of Abdominal Surgery, Geneva University Hospitals, Geneva, Switzerland; 5 Department of Visceral Surgery and Medicine, Inselspital, Bern University Hospital, University of Bern, Bern, Switzerland; 6 Transplantation Center, Service of Gastroenterology and Hepatology and Service of Immunology and Allergy, Lausanne University Hospital, University of Lausanne, Lausanne, Switzerland; 7 Department of Gastroenterology and Hepatology, University Hospital Zürich, University of Zürich, Zurich, Switzerland; 8 Department of Visceral and Transplantation Surgery, University Hospital Zurich, University of Zurich, Zurich, Switzerland; 9 Department of Gastroenterology and Hepatology, Geneva University Hospitals, Geneva, Switzerland; 10 Swisstransplant, The Swiss National Foundation for Organ Donation and Transplantation, Bern, Switzerland; 11 Division of Hepatic Surgery and Liver and Kidney Transplantation, ASST Grande Ospedale Metropolitano Niguarda, Milan, Italy; 12 Ph.D. Course in Clinical and Experimental Sciences, University of Padua, Padua, Italy

**Keywords:** donation after circulatory death donor, end-ischemic hypothermic oxygenated perfusion, ischemic cholangiopathy, liver transplantation, normothermic regional perfusion

## Abstract

Advanced preservation strategies are increasingly used in liver transplantation following donation after circulatory death (DCD). This nationwide observational study evaluated the association between abdominal normothermic regional perfusion (A-NRP) and 1-year graft survival in a Swiss cohort of 254 DCD liver transplants performed between 2017 and 2023. Recovery strategies compared included A-NRP and super-rapid recovery (SRR), both predominantly followed by end-ischemic hypothermic oxygenated perfusion (HOPE). Primary endpoint was 1-year graft loss; secondary endpoints included 1-year patient mortality, primary non-function, non-anastomotic strictures (NAS), biliary and vascular complications and need for re-transplantation. Overall, 53 grafts were retrieved using A-NRP (71% followed by HOPE) and 201 using SRR (90% followed by HOPE). Compared with SRR, A-NRP grafts demonstrated lower rates of graft loss (5.7% vs. 24.6%), 1-year mortality (1.9% vs. 13.3%), NAS (5.7% vs. 20.5%), other biliary complications (1.9% vs. 12.6%) and re-transplantation (3.8% vs. 12.8%). In multivariable Cox regression analysis, A-NRP was associated with lower risk of 1-year graft loss and patient mortality after adjustment for UK DCD risk category and HOPE use. These findings suggest that A-NRP is associated with improved outcomes. Further studies are needed to define the role of *ex-situ* perfusion after A-NRP in optimizing results.

## Graphical Abstract

**Figure:**
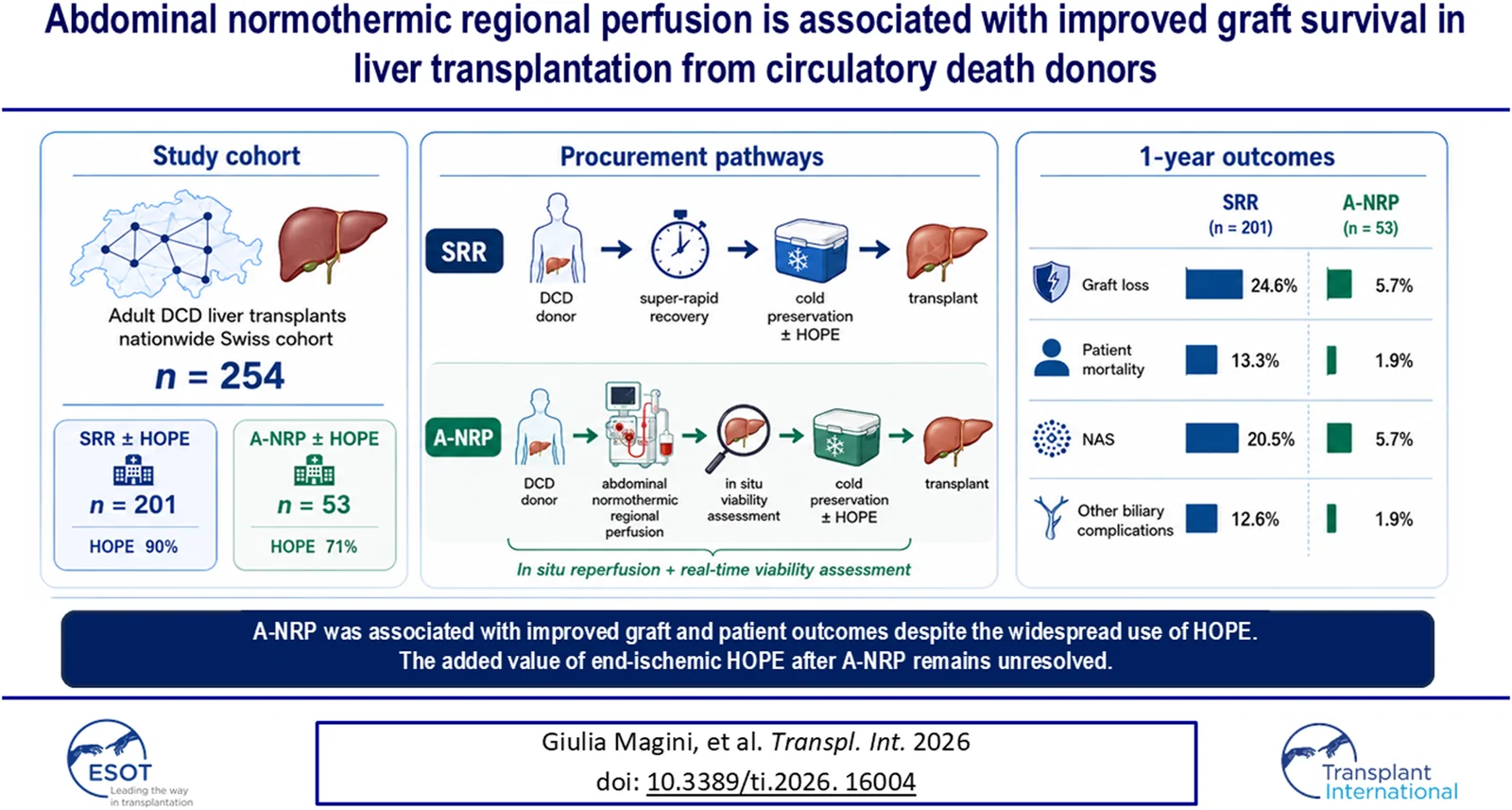

## Introduction

Donation after circulatory death (DCD) has significantly expanded the donor pool, helping to address the persistent shortage of transplantable organs.

However, early experiences using super-rapid recovery (SRR) and static cold storage (SCS) yielded suboptimal outcomes, with higher rates of biliary complications and inferior graft survival compared with grafts from donation after brain death (DBD) [1, 2]. These inferior results were largely attributed to the additional functional warm ischemia time (fWIT) inherent to DCD, which exacerbates ischemia-reperfusion injury, particularly when combined with additional risk factors such as cold ischemia time (CIT) > 6 h, donor age > 50 years, macrovesicular steatosis > 30%, donor body mass index (BMI) > 30 kg/m^2^ and complex recipient profiles (e.g., Model for End-Stage Liver Disease (MELD) score > 25 points, portal vein thrombosis or re-transplantation) [3].

The implementation of more restrictive selection criteria and predictive tools such as the UK DCD Risk Score [4], has enabled safer use of DCD grafts with results approaching those of DBD transplantation [5].

More recently, the introduction of advanced perfusion strategies including *in-situ* abdominal normothermic regional perfusion (A-NRP) and *ex-situ* preservation techniques such as normothermic machine perfusion (NMP) and hypothermic oxygenated machine perfusion (HOPE), has improved graft viability, thereby permitting broader utilization of marginal DCD donors [6–10].

In Switzerland, DCD donors currently represent nearly 35% of all liver transplants and this proportion is steadily increasing [11]. While HOPE has become the standard for *ex-situ* preservation, A-NRP is not uniformly implemented across procurement centers, with SRR still being the predominant retrieval technique for DCD livers.

This nationwide study compares outcomes of DCD liver transplantation using A-NRP versus conventional SRR, with both approaches most often combined with end-ischemic HOPE. By auditing real-world practice, we aim to provide evidence to inform future standardization of retrieval and preservation and offer practical insights for centers considering a transition to NRP and perfusion-based preservation strategies.

## Materials and methods

### Study design and population

This observational cohort study included all adult DCD liver transplantations performed in Switzerland between 1 January 2017, and 31 December 2023, with outcomes evaluated through to 31 December 2024.

Switzerland comprises 10 procurement hospitals and three liver transplantation centers. Each donor hospital applied its own procurement strategy for abdominal organ recovery, either SRR or A-NRP. During the study period, A-NRP was performed exclusively at one of the three Swiss transplant centers. Livers recovered under A-NRP were subsequently allocated among the three centers and, in selected cases, transferred to collaborating transplant programs in northern Italy through the established Swiss–Italian organ sharing agreement. In both procurement settings, use of HOPE was protocolized and performed as part of routine practice. Its omission in some cases was solely due to logistical constraints rather than based on selective, case-by-case clinical judgment or graft-related factors.

At most centers, graft acceptance was guided by the UK DCD Risk Score [4] with predefined rejection thresholds set at > 10 points for externally retrieved donors and at > 11 for local donors. Donors older than 70 years were deemed ineligible. In contrast, one center primarily based graft acceptance on *ex-situ* viability assessment during HOPE [12] without age restrictions. Liver biopsy was performed in cases where there was concern regarding donor marginality based on donor comorbidities. A cut-off of 30% macrovesicular steatosis was adopted by the centers as a criterion for organ refusal.

For grafts recovered using A-NRP, *in-situ* viability was assessed according to the biochemical criteria detailed in the “A-NRP Biochemical Assessment” section.

At the time of listing, each transplant center independently evaluated recipient eligibility for DCD liver transplantation according to local clinical protocols.

If the graft was declined by all Swiss centers according to local acceptance criteria, it was offered to Italian partner centers through the established Swiss–Italian organ sharing agreement, where final acceptance was determined according to the receiving center’s own clinical assessment and allocation framework. The study was conducted in accordance with the Declaration of Helsinki and the Declaration of Istanbul and was approved by the Institutional Ethics Committee of Geneva (BASEC 2022-00515). Recipient-level outcome data from grafts transplanted in Italy were provided by the collaborating Italian transplant program in accordance with its local ethics-approved retrospective liver transplant registry and recipient consent framework. Italian recipients had provided informed consent for data collection and research use, including DCD liver transplantation and machine perfusion. Data shared for the present analysis were coded and contained no directly identifying information (Registry number: 202-30032022).

### Data sources and variables

Recipient data were extracted from the Swiss Transplant Cohort Study (STCS–FUP 173) database, while donor-related data were obtained from the Swiss Organ Allocation System (SOAS). Missing data were retrieved directly from transplant centers.

The following donor variables were recorded: age, sex, BMI. Graft-specific parameters included functional warm ischemia time (fWIT), defined according to Swiss national DCD standards, as the interval between sustained donor MAP <50 mmHg and cold perfusion in SRR or initiation of A-NRP in perfused donors; peripheral oxygen saturation was not included in the definition of fWIT under national guidelines during the study period. Cold ischemia time (CIT) was defined as the period between the *in-situ* cold flush of the donor liver and portal vein unclamping in the recipient. Time spent on machine perfusion was not included in the CIT calculation. Recipient variables included: age, sex, BMI, laboratory MELD score at the time of transplantation and the UK DCD Risk Score [4]. Post-transplant outcomes were assessed at 1 year and included primary non-function (PNF), non-anastomotic biliary strictures (NAS), anastomotic strictures (AS), other biliary complications, arterial complications, re-transplantation, and patient death.

PNF was defined as irreversible graft failure leading to re-transplantation or death within the first 7 days post-transplantation. NAS was defined by the presence of a patent hepatic artery associated with clinical and biochemical signs of cholestasis, and imaging evidence of intra- and/or extrahepatic biliary strictures [13].

### Study end points

The primary endpoint of this study was 1-year graft loss, defined as graft failure resulting in re-transplantation or patient death within 1 year after transplantation. This outcome was compared between recipients of liver grafts recovered via A-NRP with or without HOPE and those retrieved using SRR with or without HOPE.

Secondary endpoints included all-cause mortality within 1 year post-transplantation, PNF, biliary complications (including both non-anastomotic and anastomotic biliary strictures, bile duct stones or casts and biliary leaks), hepatic artery thrombosis (HAT) and hepatic artery stenosis (HAS) and re-transplantation, within 1 year of the index procedure.

### DCD recovery methods

#### DCD donor preparation

The process of DCD donor identification is described in the Supplementary Material S1. On the day of the procedure, the donor was transferred to a dedicated room for therapeutic withdrawal. Fifteen minutes before extubation, intravenous heparin (300 IU/kg) was administered. A sustained drop in mean arterial pressure below 50 mmHg for more than 2 min marked the onset of the fWIT. Cardiac arrest was confirmed by electrocardiographic asystole and the absence of cardiac motion on transthoracic echocardiography (TTE). A mandatory 5-min “no-touch” period was observed. Once death was legally declared, organ recovery was initiated using one of two techniques: SRR or A-NRP.

#### Super-rapid recovery (SRR)

A midline laparotomy was performed. The thoracic cavity was not routinely opened before initiation of abdominal cold perfusion for liver recovery. The distal abdominal aorta was cannulated, the supra-celiac aorta was clamped, and cold perfusion was initiated using a 4 °C preservation solution, vented through the inferior vena cava. This step marked the end of the warm ischemia period and the start of cold ischemia. The abdominal cavity was then packed with sterile ice, and the liver was recovered as quickly as possible according to standard surgical protocols.

#### Abdominal normothermic regional perfusion (A-NRP)

The procedure was explained to the family on the day preceding retrieval, and consent was obtained. Since 2021, femoral vessel catheters have been placed prior to death confirmation under ultrasound guidance. Once cardiac arrest occurred, family members present were accompanied out of the unit by a staff member, and the donor was transferred to a nearby room pre-equipped for A-NRP. After legal confirmation of death, the ICU team performed cannulation for A-NRP, initially under ultrasound guidance, visualizing the inferior vena cava and abdominal aorta for guidewire placement, cannula insertion and occlusion balloon placement, and, since 2022, under fluoroscopic guidance.

Once vascular access was established and the occlusion balloon was correctly positioned and inflated, no surgical clamping of the aorta was performed, and no venting procedure was used. Correct balloon positioning and complete exclusion of cerebral reperfusion were verified by continuous invasive monitoring of the radial arterial pressure, which was required to remain <20 mmHg throughout A-NRP.

A-NRP was then initiated. Target parameters included a blood flow of 3–4 L/min, MAP 50–70 mmHg, temperature of 36 °C–37 °C, abdominal PaO_2_ > 20 kPa, and PaCO_2_ 4–5 kPa. Hemoglobin level was maintained > 60 g/L, with red blood cell transfusions as required. Blood pressure and A-NRP flow were maintained with norepinephrine and 0.9% NaCl fluid infusions. Arterial blood gases were analyzed every 30 min from A-NRP initiation and hepatic transaminases, and Factor V were measured hourly.

After typically 90–120 min of A-NRP, the donor was transferred to the operating room for organ recovery. Hepatectomy was performed according to standard surgical protocols. Once the liver was ready for retrieval, A-NRP was discontinued, and the same circuit was used for cold perfusion (4 °C) with IGL® solution.

#### A-NRP biochemical assessment

Arterial blood gas analysis was obtained at the onset of A-NRP and repeated every 30 min until the start of cold perfusion with IGL® solution. Liver transaminases (AST and ALT) were measured after the first hour of A-NRP and subsequently every hour until the termination of the perfusion.

Criteria for early termination of the A-NRP procedure were predefined in the institutional protocol. Hepatic perfusion was considered inadequate in the presence of a sustained increase in transaminase levels and/or the absence of a clear downward trend in arterial lactate concentration during perfusion. Because no validated thresholds for transaminases and lactate dynamics during A-NRP exist in the published literature, decisions were based on the overall biochemical evolution rather than on fixed numerical cut-offs.

#### Organ transport and post-recovery protocol

Regardless of the procurement technique, all liver grafts were placed in sterile, temperature-controlled transport containers and transferred to the recipient center under cold preservation conditions. In most cases, the graft subsequently underwent HOPE for approximately 120 min before implantation.

#### *Ex-vivo* HOPE

Following back-table preparation, HOPE was performed using the Liver Assist® device (Organ Assist B.V., Groningen, Netherlands) in two centers and the VitaSmart™ (Bridge to Life™ Ltd., CE-marked) in one center. The use of HOPE was protocolized across all three participating centers and was performed as part of routine practice. Its omission in some cases was solely due to logistical constraints; otherwise, it was systematically applied rather than based on selective, case-by-case clinical judgment or graft-related factors. *Ex-situ* liver perfusion was performed via the portal vein only, under active oxygenation and controlled low-pressure and low-flow (3 mmHg, 150–250 mL/min). All HOPE procedures in this study utilized the University of Wisconsin Machine Perfusion solution (Belzer MPS, Bridge to Life™) as perfusate. In one center, liver graft quality was additionally assessed after 30 min of perfusion by fluorescence spectroscopic analysis of the perfusate, as previously described [2].

### Statistical analysis

Patients were categorized according to the organ procurement technique: SRR or A-NRP. Categorical variables were reported as absolute counts (n) and percentages (%), while continuous variables were expressed as medians with interquartile ranges (IQR). Comparisons between groups were performed using the Chi-squared or Fisher exact tests for categorical variables as appropriate, and the Mann-Whitney U test was used for continuous variables. One-year patient survival and graft loss were estimated using the Kaplan-Meier method and compared using a log-rank test. To evaluate the association between the use of A-NRP (compared with SRR) and the risk of graft loss and mortality within 1-year, a Cox proportional hazards regression model was fitted. Because organ preservation method (HOPE vs. SCS) differed between procurement strategies and may independently influence graft outcomes, we treated preservation method as a separate binary covariate in all multivariable models, together with the UK DCD Risk Score, which was specified *a priori*. Given the limited number of outcome events, multivariable adjustment was intentionally restricted to clinically prespecified covariates rather than based on univariable screening, to reduce the risk of overfitting. A two-sided *p*-value ≤ 0.05 was considered statistically significant. As a sensitivity analysis, we performed a propensity score–based inverse probability of treatment weighting (IPTW) analysis. The propensity score estimated the probability of receiving A-NRP using HOPE use, UK DCD risk category and transplant era as a proxy for temporal learning-curve effects. Stabilized weights were applied in Cox proportional hazards models with robust standard errors. Covariate balance before and after weighting was assessed using standardized mean differences and displayed in a love plot. All statistical analyses were performed with Stata version 16.0 (StataCorp, College Station, TX, USA).

## Results

### Patient characteristics

This nationwide cohort included 262 adult liver transplants from DCD donors performed between 2017 and 2023. Of these, 8 patients were excluded from the analysis due to missing data and/or absence of informed consent. Of the remaining 254, 201 grafts (79.1%) were procured using SRR and 53 (20.9%) using A-NRP. Additional HOPE was applied in 181 grafts of the SRR group (90%), and 38 cases (71.7%) of the A-NRP group (*p* < 0.001) (Table 1).

**TABLE 1:** Patient characteristics.

N = 254	SRR (n = 201)	A-NRP (n = 53)	*p*
Donor characteristics			
Age, median (IQR)	60 (49.0–71.0)	61 (52.0–69.0)	0.892
BMI, median (IQR)	25.7 (23.6–27.8)	26 (23.0–29.4)	0.954
Hepatectomy time, (median IQR)	41.5 (29.7–55.0)	45.5 (36.0-54.5)	0.178
Recipient characteristics			
Age, median (IQR)	60 (53.0–64.0)	60 (55.6–64.0)	0.430
Sex, Male n (%)	159 (79.0%)	44 (83.0%)	0.66
MELD, median (IQR)	12 (8–19)	11 (8–18)	0.528
Surgery details			
Transplant center, n (%) -Bern -GE -ZH -Italy	32 (15.9%) 47 (23.4%) 122 (60.7%) 0 (0%)	15 (28.3%) 12 (22.6%) 20 (37.7%) 6 (11.3%)	<0.01
HOPE, n (%)	181 (90.0%)	38 (71.7%)	<0.001
Functional warm ischemia, median (IQR)	24 (18–29)	26 (22–29)	0.155
Total cold ischemia time (minutes), median (IQR)	370 (270–474)	377 (317–480)	0.211
UK DCD risk score, median (IQR)	8 (6–10)	8 (6-10)	0.429
UK DCD risk group, n (%) -Futile -High risk -Low risk	49 (24.4%) 108 (53.7%) 44 (21.9%)	14 (26.42%) 31 (58.5%) 8 (15.1%)	0.552

Chi-2, or Fisher-exact for categorical variables, Wilcoxon Ranksum test for continuous variables.

All A-NRP procedures considered for liver recovery were successfully completed, with no conversion to SRR. No A-NRP graft was excluded or reclassified because of technical failure, inadequate regional perfusion, or unfavorable biochemical evolution during perfusion.

Donor characteristics were comparable between groups. Median donor age was 60 years (IQR 49–71) in the SRR group and 61 years (IQR 52–69) in the A-NRP group. Median BMI was 25.7 kg/m^2^ (IQR 23.6-27.8) and 26 kg/m^2^ (IQR 23.0–29.4), respectively.

Median fWIT did not differ significantly between groups, 24 min (IQR 18–29) for SRR and 26 min (IQR 22–29) for A-NRP (*p* = 0.155). Similarly, total CIT was comparable: 370 min (IQR 270-467) for SRR and 377 (325-480) for A-NRP (*p* = 0.211).

Recipient demographics were also well balanced. The majority were male (79% in the SRR group and 83% in the A-NRP group, *p* = 0.66), with a median age of 60 years in both groups (*p* = 0.403). Median MELD score at transplantation was 12 (IQR 8–19) for SRR and 11 (IQR 8–18) for A-NRP (*p* = 0.528). The median UK DCD Risk Score was 8 (IQR 6–10) in both groups (*p* = 0.429). In both groups, approximately 25% of grafts were classified as futile, despite two of the centers having set a cutoff of 10 for organ acceptance.

Most transplants were performed at the largest participating center (55.9%), followed by two medium-sized centers (23.2% and 18.5%). A small proportion of recovered organs (2.3%) included in the present study were ultimately transplanted abroad.

### Patient outcomes

At 1-year post-transplantation, 20.8% (53/254) of patients experienced graft loss, 17.3% (44/254) developed NAS, 10.2% (26/254) presented with other biliary complications, 5.9% (15/254) developed PNF, 11% (28/254) required re-transplantation, and 11% (28/254) ultimately died.

Across all major outcomes, adverse events were more frequent among recipients of grafts procured via SRR compared to those recovered using A-NRP (Table 2).

**TABLE 2:** Patient outcomes.

N = 254	SRR (n = 201)	A-NRP (n = 53)	p
Primary non-function, n (%)	13 (6.40%)	2 (3.77%)	0.468
Graft loss at 1-year, n (%)	50 (24.63%)	3 (5.66%)	0.002
Biliary non-anastomotic stenosis, n (%) - 3 missing (3 SRR)	41 (20.50%)	3 (5.66%)	0.011
Other biliary complications, n (%) – 4 missing (4 SRR)	25 (12.56%)	1 (1.89%)	0.023
Hepatic artery thrombosis, n (%) – 1 missing (1 A-NRP)	6 (2.96%)	2 (3.85%)	0.742
Hepatic artery stenosis, n (%)	11 (5.42%)	1 (1.89%)	0.279
Re-transplantation, n (%)	26 (12.81%)	2 (3.77%)	0.061
Death at 1-year, n (%)	27 (13.30%)	1 (1.89%)	0.018

Fisher exact test for categorical variables.

One-year graft loss occurred in 24.6% of SRR +/- HOPE cases (of which 17% were attributable to PNF or NAS), versus 5.7% in the A-NRP +/- HOPE group (all due to PNF or NAS; *p* = 0.002).

NAS were observed in 20.5% of SRR recipients versus 5.7% in the A-NRP group (*p* = 0.011) (Supplementary Figure S1), while other biliary complications affected 12.6% of SRR grafts compared to 1.89% of A-NRP grafts (*p* = 0.023).

The need for re-transplantation was higher in the SRR group (12.8%) compared with the A-NRP group (3.8%), though this difference did not reach statistical significance (*p* = 0.061). One-year mortality was significantly higher among SRR recipients (13.3%) than among A-NRP recipients (1.9%, *p* = 0.018).

**FIGURE 1:**
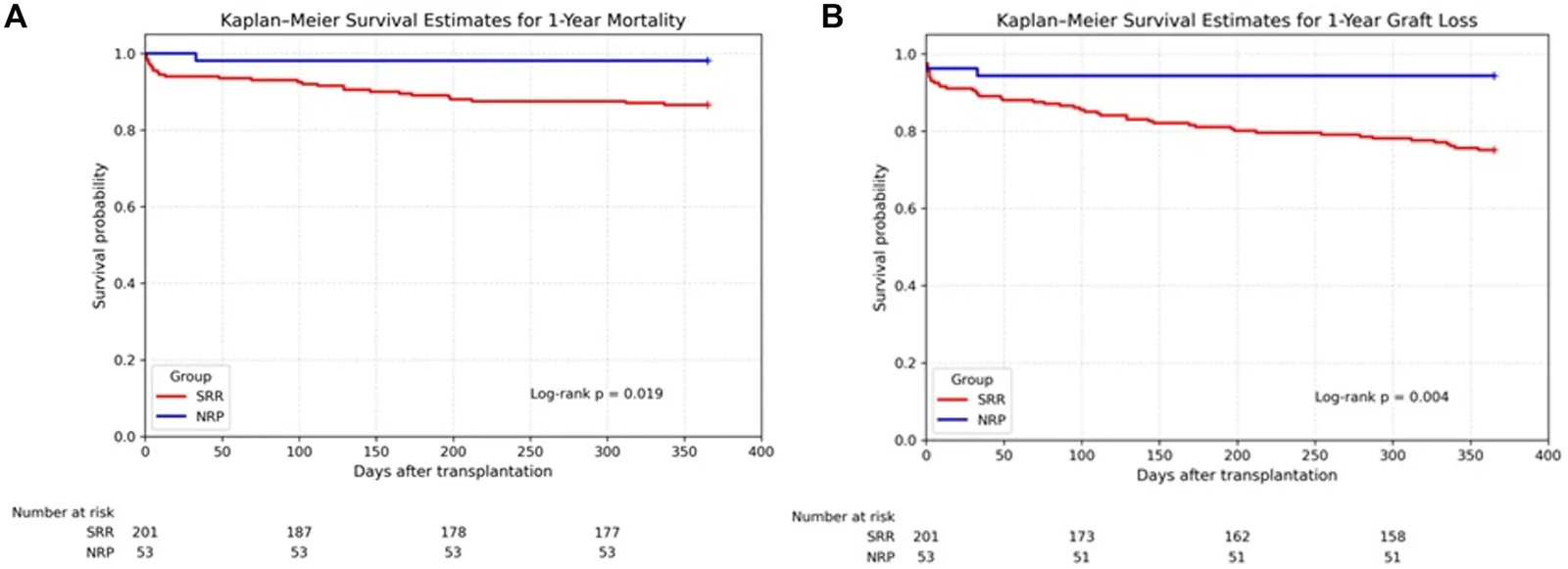
Kaplan–Meier survival estimates according to procurement technique. **(A)** One-year patient survival. **(B)** One-year graft survival. Comparisons between A-NRP and SRR procurement techniques were performed using the log-rank test. *Two-column fitting image.*

Kaplan–Meier survival analysis demonstrated significantly improved graft and patient survival in the A-NRP group compared to SRR (Figure 1). In multivariable Cox regression analysis adjusted for UK DCD Risk Scoreand for the use of HOPE (Table 3), A-NRP was associated with a lower risk of 1-year graft loss (HR 0.20, 95% CI 0.06–0.67, *p* = 0.008) and 1-year mortality (HR 0.11, 95% CI 0.02–0.85, *p* = 0.034). In the propensity score–based IPTW sensitivity analysis including HOPE use, UK DCD risk category, and transplant era, A-NRP remained associated with a lower risk of 1-year graft loss (HR 0.28, 95% CI 0.08–0.95, *p* = 0.040). The mortality estimate remained directionally consistent with the primary analysis but was no longer statistically significant (HR 0.21, 95% CI 0.03–1.56, *p* = 0.129), reflecting the limited number of mortality events in the A-NRP group (Supplementary Table S1). Covariate balance improved after IPTW, although residual imbalance persisted for transplant era (Supplementary Figure S2).

**TABLE 3:** Survival analysis.

N = 254	HR for 1-year death (95% CI)	*p*
Univariable		
A-NRP	0.13 (0.02-0.98)	0.047
Multivariable		
A-NRP HOPE (yes) UK DCD classification Low-risk High risk Futile	0.11 (0.02-0.85) 0.60 (0.20-1.77) Reference 1.41 (0.46-4.33) 2.81 (0.88-8.95)	0.034 0.356 0.551 0.080

N = 254	HR for 1-year graft loss (95% CI)	*p*
Univariable		
A-NRP	0.21 (0.07-0.67)	<0.01
Multivariable		
A-NRP HOPE (yes) UK DCD classification Low-risk High risk Futile	0.20 (0.06-0.67) 0.96 (0.41-2.29) Reference 1.18 (0.57-2.43) 1.27 (0.56-2.86)	<0.01 0.941 0.645 0.571

Cox regression analysis.

### Organ utilization rate

The exploratory organ utilization analysis is presented in the Supplementary Material S2. Importantly, no organ was discarded because of the use of A-NRP itself or because of an A-NRP-related technical or perfusion complication.

### Trend of biochemical biomarkers during A-NRP

Supplementary Figures S3, S4 show the trends of biochemical biomarkers during A-NRP stratified by transplant outcomes and UK DCD Risk Score. Notably we observed a modest influence of ‘donor quality’ stratified according to UK DCD Risk Score on biomarkers during perfusion, as well as weak stratification according to outcomes.

## Discussion

In this nationwide Swiss multicenter cohort study, we compared outcomes of DCD liver transplantation following A-NRP versus conventional SRR, both predominantly combined with HOPE. To our knowledge, this is one of the largest contemporary analyses on this topic and uniquely includes detailed documentation of HOPE utilization. Consistent with previous studies and recent meta-analyses, A-NRP was associated with a reduced risk of 1-year graft loss compared with SRR, mainly driven by lower rates of PNF and NAS [9, 14, 15]. This association remained significant after adjustment for HOPE use and UK DCD Risk Score and was further supported by an IPTW sensitivity analysis accounting for HOPE use, UK DCD risk category, and transplant era.

When benchmarked against internationally accepted outcome thresholds for DCD liver transplantation [16], A-NRP-retrieved grafts met expectations, with 1-year graft loss and NAS rates of 5.7%, and a 1-year patient mortality of 1.9%. In contrast, SRR +/- HOPE exceeded benchmark thresholds across all parameters. These poorer outcomes may partly reflect the early phase of our DCD program, when extended-criteria grafts were used during the early learning curve. These results are less favorable than the Schlegel benchmarks, which are based on low-risk DCD populations and experience from high-volume centers [16].

A-NRP was associated with lower 1-year graft loss and patient mortality in the primary multivariable analysis adjusted for HOPE and the UK DCD Risk Score. This association was further explored in the IPTW sensitivity analysis including HOPE use, UK DCD risk category, and transplant era, in which the association with lower graft loss remained statistically significant, whereas the association with mortality remained directionally consistent but lost statistical significance, likely due to the low number of deaths in the A-NRP group. These findings should therefore be interpreted as supportive rather than definitive evidence of an independent effect of A-NRP. Together, they support further structured implementation and prospective evaluation of A-NRP within standardized DCD programs.

Whether end-ischemic HOPE following A-NRP provides additional benefit remains uncertain. Recent multicenter observational data suggest a potential synergistic effect [17]. In addition, national data from Italy, where the combination of A-NRP and HOPE is routinely used, reported lower rates of post-transplant acute kidney injury [18].

Several European countries, including France, Italy, and Norway, have integrated A-NRP into national DCD procurement pathway [19] while Spain increasingly favors A-NRP over SRR [8]. In contrast, North American experience remains limited, with only a few pioneering centers having developed A-NRP programs [14, 20]. Unlike much of the existing literature, often derived from high-volume expert centers with highly selective donor practices, our study reflects real-world conditions in which both SRR and A-NRP are actively practiced, enhancing the external validity of our findings.

A major advantage of A-NRP over SRR is the ability to enable real-time functional graft assessment during perfusion. By restoring oxygenated blood flow after circulatory arrest, A-NRP creates a physiological window for *in-situ* viability assessment. Although recent ELITA consensus guidelines acknowledge this advantage, substantial variability persists in the viability parameters used, and no universally accepted thresholds exist [21]. According to ELITA consensus recommendations, key assessment parameters during A-NRP should include macroscopic appearance, transaminase kinetics, and lactate clearance [21]. Emerging biomarkers during *ex-situ* perfusion such as flavin mononucleotide (FMN), a marker of mitochondrial injury, may further refine viability assessment [22, 23].

Several limitations must be acknowledged. Causal inference is limited by the observational design and non-random allocation of procurement strategies, with potential residual confounding despite adjustment. Randomized evaluation is challenging in this setting, where procurement and allocation strategies are increasingly individualized and risk-adapted [24]. A-NRP was routinely performed in only one national DCD procurement center; therefore, center-, team-, era-, donor-selection, preservation-strategy, and HOPE-related effects cannot be fully disentangled from the effect of A-NRP, despite national allocation of A-NRP-retrieved grafts. Although transplant era was included in the IPTW analysis to partly account for learning-curve effects, residual temporal and practice-related confounding cannot be excluded. Survival analyses were limited by the low number of events in the A-NRP cohort, particularly for mortality, resulting in wide confidence intervals. This was reflected in the IPTW sensitivity analysis, in which the mortality estimate remained directionally consistent but was no longer statistically significant. Follow-up was limited to 1 year, precluding assessment of longer-term outcomes. Finally, although the present study focused on liver outcomes, A-NRP should not be regarded as a liver-directed intervention only. By restoring abdominal circulation, A-NRP may also affect the assessment, utilization, and outcomes of kidneys and pancreas, as well as the coordination of thoracic recovery workflows when lungs or heart are considered. Non-liver recipient outcomes were not evaluated, but no organ was discarded because of an A-NRP-related technical or perfusion complication. Future national studies should evaluate the broader impact of A-NRP on overall organ utilization, non-liver graft outcomes, logistics, and costs.

Despite these limitations, this nationwide study provides important insights for the development of DCD liver transplantation programs. The favorable outcomes associated with A-NRP support further structured implementation and prospective evaluation within standardized DCD pathways. The study also highlights the need for harmonized procurement and preservation protocols and structured viability assessment criteria to facilitate the dissemination of best practices. Whether grafts recovered using A-NRP derive additional benefit from end-ischemic HOPE remains unresolved and warrants prospective investigation. Collectively, these measures may improve organ utilization, reduce discards, and optimize long-term outcomes in DCD liver transplantation.

## Data Availability

The raw data supporting the conclusions of this article will be made available by the authors, without undue reservation.
