# Supplementary material for: Abdominal normothermic regional perfusion is associated with improved graft survival in liver transplantation from circulatory death donors
Source: Transpl Int. 2026 Aug 26;39:16004. doi: 10.3389/ti.2026.16004 (PMC13557021; doi:10.3389/ti.2026.16004)
Supplement: Supplementary file 1 [file Supplementaryfile1.docx]

**SUPLEMENTARY MATERIAL**

**S1 DCD Donor Identification**

Potential donation after circulatory death (DCD) donors were identified among intensive care unit (ICU) patients receiving mechanical ventilation for whom a decision to withdraw life-sustaining treatment had been made. Once this decision was jointly confirmed by the medical team and the next of kin, the presumed wishes of the patient regarding organ donation were assessed based on an official donor card, advance directives, or family testimony, in close collaboration with the local organ and tissue donation team.

The timing of withdrawal of life-sustaining treatment was scheduled in agreement with the family. Pre-procurement evaluation of organ suitability included routine laboratory testing and contrast-enhanced computed tomography angiography (CTA), the results of which were uploaded to the Swiss Organ Allocation System (SOAS), managed by Swisstransplant. The DCD procedure proceeded only if at least one organ was successfully allocated to a recipient through the SOAS.

## S2 Organ Utilization Rate

## In the exploratory analysis, considering all organs offered and transplanted during the study period, the organ utilization rate was 75.3% for A-NRP (55 transplanted organs out of 73 offered) compared with 66.5% for SRR (207 transplanted organs out of 311 offered). The difference between groups did not reach statistical significance (*p* = ns). Among A-NRP donors, livers were declined because of donor or graft marginality criteria including comorbidities, steatosis on biopsy, anatomical or logistical considerations, or absence of a suitable compatible recipient. No organ was discarded because of an A-NRP-related technical or perfusion complication. The discrepancy between the reported organ acceptance rates and the number of patients included in the comparison is due to the exclusion of eight patients because of missing data.

## S3 Trend of biochemical biomarkers during A-NRP

**Figure S3** and **Figure S4** show trend of biochemical biomarkers during A-NRP stratified by transplant outcomes and UK DCD score. Notably we observed a modest influence of ‘donor quality’ stratified according to UK DCD score on biomarkers during perfusion, as well as weak stratification according to outcomes. During the study period, no liver was declined during A-NRP due to insufficient metabolic response, including absence of lactate improvement.

**Figure S1**

**Figure S1. Incidence of ischemic cholangiopathy and biliary complications in the A-NRP and SRR groups.**

Proportion of recipients with ischemic-type cholangiopathy and other biliary complications—including anastomotic strictures, biliary leaks, and bile duct stones/casts—comparing the normothermic regional perfusion (A-NRP) and super-rapid recovery (SRR) cohorts.

**Figure S2 Love plot**

**Figure S2.**

Love plot showing covariate balance before and after IPTW. Covariate balance improved after weighting, with most standardized mean differences below 0.10. A small residual imbalance persisted for transplant era.

**Figure S3**

**Figure S3. Evolution of key biochemical biomarkers during abdominal normothermic regional perfusion (A-NRP) according to transplant outcomes.**

Serial changes in lactate, aspartate aminotransferase (AST), alanine aminotransferase

(ALT), and Factor V levels throughout A-NRP, stratified by subsequent graft outcome.

**Figure S4**

**Figure S4. Evolution of key biochemical biomarkers during abdominal normothermic regional perfusion (A-NRP) according to UK DCD Risk Score categories.**

Serial changes in key biochemical markers measured in the blood of reperfused organs during A-NRP, stratified by UK DCD Risk Score categories, illustrating the relationship between donor risk profile and metabolic recovery.

**Table S1 IPTW Cox regression models**

| Outcome  N=247 | HR for A-NRP vs SRR | 95% CI | *p* |
| --- | --- | --- | --- |
| One-year patient mortality | 0.21 | 0.03–1.56 | 0.127 |
| One-year graft loss | 0.28 | 0.08–0.95 | 0.038 |

**Table S1. IPTW Cox regression models.**

The propensity score estimated the probability of receiving A-NRP using HOPE use, UK DCD risk category, and transplant era. Stabilized weights were applied in marginal Cox proportional hazards models with robust standard errors.

**Capsule Sentence Summary**

In a nationwide Swiss DCD liver transplant cohort, abdominal normothermic regional perfusion was associated with lower one-year graft loss and fewer biliary complications despite widespread HOPE use.
